# Supplementary material for: Inulin Supplementation Disturbs Hepatic Cholesterol and Bile Acid Metabolism Independent from Housing Temperature
Source: Nutrients. 2020 Oct 20;12(10):3200. doi: 10.3390/nu12103200 (PMC7589137; doi:10.3390/nu12103200)
Supplement: Supplementary file 1 [file nutrients-12-03200-s001.pdf]

Supplementary Table 1

Supplementary Table 1. Composition of the Inulin and control diet.

	S5714-E710	S5714-E716
**Proximate contents**	**CD**	**ICD**
Carbohydrates	64,3	30, 8
Crude Protein	21	21
Crude Fat	5,1	5,1
Crude fiber	0,2	33,2
**Ingredients** %		
Inulin	0	30
Cellulose	0.2	5
Starch	33	12
Maltrodextrin	19,9	6,1
**Energy** (MJ/kg)	16,2	10,7
kcal%		
Fat	12	18,4
Protein	22	33,3
Carbohydrates	66	48,3

Supplementary Figure 1

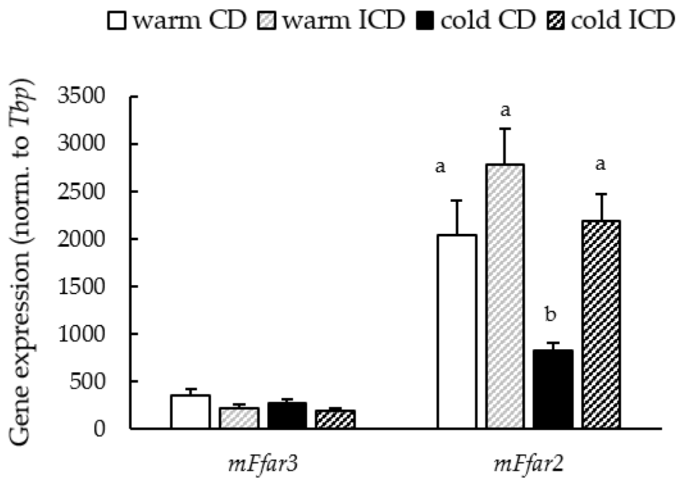

Supplementary Figure 1. Inulin does not affect expression of GPR41 and GPR43 in colon tissues. Gene expression of GPR41 (encoded by *Ffar3*) and GPR43 (encoded by *Ffar2*) in colon tissues. Levels are normalized to *Tbp* as housekeeper. Data are shown as mean values ± SEM, different letters indicate significant differences between groups ($p < 0.05$) determined by two-way ANOVA. CD warm: n=6, ICD warm: n=6, CD cold: n=6, ICD cold: n=4

Supplementary Figure 2

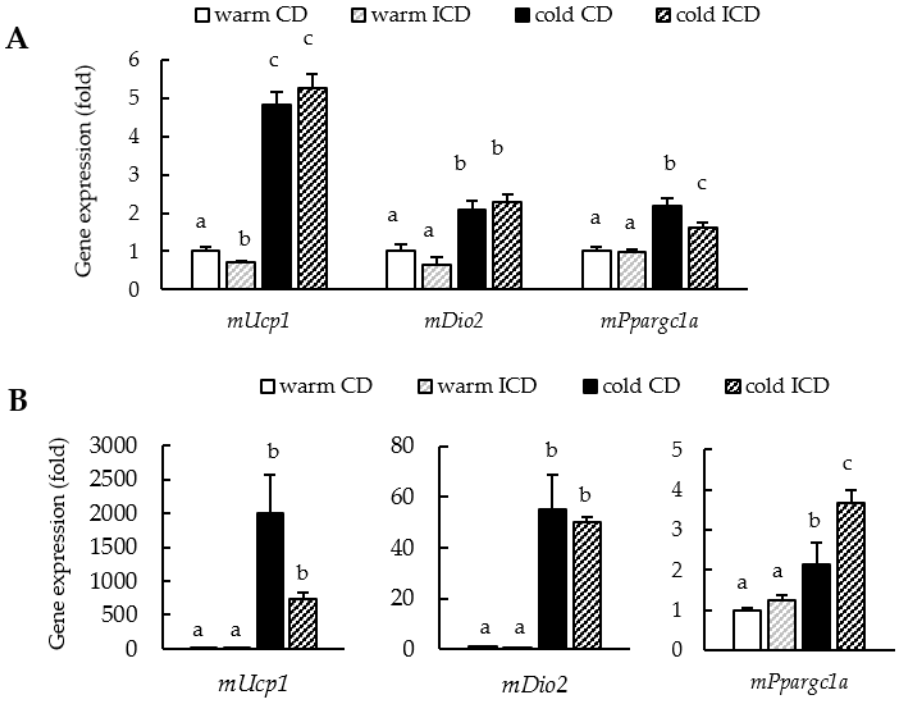

Supplementary Figure 2. Inulin does not affect BAT and ingWAT gene expression Relative gene expression of BAT marker genes in (**A**) BAT and (**B)** ingWAT. Levels are normalized to *Tbp* as housekeeper. Data are shown as mean values ± SEM, different letters indicate significant differences between groups ($p < 0.05$) determined by two-way ANOVA. CD warm: n=6, ICD warm: n=6, CD cold: n=6, ICD cold: n=5
